# Association of Vitamin D Deficiency and Poor Glycemic Control in Patients With Type 2 Diabetes Mellitus

**DOI:** 10.7759/cureus.117342

**Published:** 2026-10-02

**Authors:** Muhammad Usama, Hafiza Sidra, Muhammad Fahad Munir, Hafiz Ali Shabbir Rajput, Farah Gul, Tabish Sattar, Uzair Ullah Qureshi, Syed Hamza Elahi

**Affiliations:** 1 Medicine and Surgery, Khyber Medical University, Peshawar, PAK; 2 Internal Medicine, Lady Reading Hospital, Peshawar, PAK; 3 Internal Medicine, University of Health Sciences, Lahore, PAK; 4 Internal Medicine, Capital Medical University, Beijing, CHN; 5 Surgery, Arrowe Park Hospital, Upton, GBR; 6 Pharmacology, Medicinal Botanic Centre, Pakistan Council of Scientific and Industrial Research Labs Complex, Peshawar, PAK; 7 Internal Medicine, Hayatabad Medical Complex, Peshawar, PAK; 8 Internal Medicine, Abbottabad International Medical Institute, Abbotabad, PAK; 9 Internal Medicine, Rehman Medical College, Peshawar, PAK

**Keywords:** 25-hydroxyvitamin d, diabetes mellitus, glycemic control, hba1c, type 2 diabetes mellitus, vitamin d deficiency

## Abstract

Background: Vitamin D deficiency is common among patients with type 2 diabetes mellitus and may be associated with inadequate glycemic control.

Objective: To determine the association between vitamin D deficiency and poor glycemic control among adults with type 2 diabetes mellitus.

Materials and methods: This cross-sectional study was conducted at Lady Reading Hospital (LRH), Peshawar, Pakistan, from March 2024 to February 2025 among 350 adults with type 2 diabetes mellitus. Serum 25-hydroxyvitamin D (25(OH)D) and glycated hemoglobin (HbA1c) measurements were obtained from hospital laboratory records. Serum 25(OH)D was measured using an enzyme-linked immunosorbent assay (ELISA), according to the laboratory standard operating procedure. Vitamin D deficiency was defined as a serum 25(OH)D level <20 ng/mL and poor glycemic control was defined as HbA1c ≥7.0%. Data were analyzed using descriptive statistics, the chi-square test, independent-samples t-test, and binary logistic regression. Multivariable binary logistic regression was performed to assess the independent association between vitamin D deficiency and poor glycemic control after adjustment for age, BMI, diabetes duration, hypertension, dyslipidemia, physical inactivity, dietary adherence, medication adherence, previous diabetes education, and smoking. Adjusted odds ratios (AORs) with 95% confidence intervals (CIs) were reported.

Results: Among 350 participants, 218 (62.29%) were vitamin D deficient and 132 (37.71%) were non-deficient. Poor glycemic control was present in 226 (64.57%) participants, while 124 (35.43%) had adequate control. Vitamin D deficiency was observed in 164 (72.57%) participants with poor glycemic control compared with 54 (43.55%) with adequate control (p <0.001). Participants with poor glycemic control had lower mean serum 25(OH)D levels than those with adequate control (16.72 ± 6.43 vs. 23.84 ± 7.16 ng/mL; p <0.001). After adjustment for the prespecified covariates listed above, vitamin D deficiency was associated with higher odds of poor glycemic control (AOR = 2.87, 95% CI: 1.76-4.68; p <0.001).

Conclusion: Vitamin D deficiency was common and was independently associated with higher odds of poor glycemic control among adults with type 2 diabetes mellitus. Given the cross-sectional design, this association should not be interpreted as causal, and prospective studies are needed to clarify the relationship.

## Introduction

Type 2 diabetes mellitus (T2DM) is a major global metabolic disorder and an increasingly important public health concern. It is characterized by chronic hyperglycemia resulting primarily from insulin resistance and progressive β-cell dysfunction [1,2]. The growing prevalence of T2DM is driven by population aging, urbanization, obesity, physical inactivity, and unhealthy dietary patterns [3]. Despite advances in pharmacological treatment and diabetes self-management, achieving and maintaining optimal glycemic control remains challenging for many patients [4]. Persistent hyperglycemia is associated with an increased risk of microvascular and macrovascular complications, including nephropathy, retinopathy, neuropathy, cardiovascular disease, and stroke. Glycated hemoglobin (HbA1c) is an established clinical measure of glycemic control and reflects average blood glucose levels over the preceding several months [5].

Vitamin D status has received increasing attention because of its potential role in glucose metabolism. In addition to established factors such as obesity, diabetes duration, dietary and medication adherence, and physical inactivity, vitamin D status has been investigated as a potentially modifiable factor associated with glycemic control [6]. Vitamin D receptors are expressed in pancreatic β-cells, skeletal muscle, adipose tissue, and other metabolically active tissues, supporting a potential role for vitamin D in insulin secretion and insulin sensitivity [7]. Vitamin D may also influence glucose metabolism through effects on inflammation and cellular signaling pathways [8]. These biological mechanisms provide a plausible basis for investigating the association between vitamin D status and glycemic control in people with T2DM, although they do not establish a causal relationship.

Vitamin D deficiency is frequently reported among patients with T2DM across different populations [9]. Several observational studies have reported an inverse association between serum 25-hydroxyvitamin D [25(OH)D] concentrations and HbA1c, although findings have not been consistent across populations [10]. Differences in geographical location, sunlight exposure, nutritional status, obesity, diabetes duration, treatment patterns, and definitions of vitamin D deficiency may contribute to variation between studies. Although both T2DM and vitamin D deficiency are important health concerns in Pakistan, evidence examining the association between vitamin D status and glycemic control among Pakistani adults with T2DM remains comparatively limited [11,12].

Therefore, examining the association between vitamin D status and glycemic control may help clarify whether vitamin D deficiency is related to poor glycemic control among adults with T2DM. The present study aimed to determine the association between vitamin D deficiency and poor glycemic control among adults with T2DM attending Lady Reading Hospital, Peshawar, Pakistan.

## Materials and methods

Study design

A hospital-based cross-sectional study was conducted to determine the prevalence of poor glycemic control and to assess its association with vitamin D deficiency among adults with T2DM. Demographic, clinical, lifestyle, and treatment-related factors potentially associated with glycemic control were also evaluated.

Study setting and duration

The study was conducted at Lady Reading Hospital (LRH), Peshawar, Pakistan, over a one-year period from March 2024 to February 2025. Participants were recruited from the outpatient Department of Endocrinology and Diabetes during the study period.

Study population

The study population comprised adults with a confirmed diagnosis of T2DM who attended the outpatient Department of Endocrinology and Diabetes at LRH during the study period. Eligible participants were recruited consecutively after providing written informed consent. Participants were included in the analytical sample when a recent HbA1c measurement and a documented serum 25(OH)D measurement were available in the hospital laboratory records. Because availability of a vitamin D measurement was an inclusion requirement, the analytical sample may differ from the broader outpatient T2DM population in whom vitamin D testing was not performed.

Eligibility criteria

Eligible participants were aged ≥18 years, had a confirmed diagnosis of T2DM for at least 6 months, and had a documented HbA1c measurement obtained within the preceding 3 months and a serum 25(OH)D measurement available in the hospital laboratory records during the study period. All participants provided written informed consent.

Patients with type 1 diabetes mellitus, gestational diabetes, or other forms of diabetes were excluded. Additional exclusion criteria were pregnancy; acute diabetic emergencies; severe illness requiring hospitalization; cognitive impairment or severe psychiatric conditions that could preclude reliable data collection; incomplete medical records; and unavailable HbA1c or serum 25(OH)D measurements.

Sample size and sampling technique

The sample size was determined using the single-population proportion formula for a cross-sectional study. Based on a previous study among patients with type 2 diabetes mellitus in Pakistan, the expected prevalence of poor glycemic control was 65.25% [12]. Using a 95% confidence level and a 5% margin of error, the minimum required sample size was calculated as approximately 349 participants and was rounded up to 350 participants. Therefore, 350 adult patients with type 2 diabetes mellitus were included in the study. Consecutive non-probability sampling was used, with eligible patients attending the Department of Endocrinology and Diabetes recruited consecutively until the required sample size was achieved. The sample size was calculated using the single-population proportion formula for a cross-sectional study:

$n = \frac{Z^2 P(1-P)}{d^2}$

$n = \frac{(1.96)^2(0.6525)(1-0.6525)}{(0.05)^2}$

$n = \frac{(3.8416)(0.6525)(0.3475)}{0.0025}$

$n = \frac{0.8707}{0.0025}$

*n = 348.28*

Data collection procedure

Data were collected using a structured, standardized, and pretested questionnaire administered through face-to-face interviews by trained investigators. Additional clinical and laboratory information was obtained from participants' medical records and hospital laboratory reports. The collected information included sociodemographic characteristics, lifestyle factors, diabetes treatment modalities, family history of diabetes, comorbidities, diabetes-related complications, physical activity, dietary adherence, medication adherence, and diabetes self-management practices.

Height and weight were measured using standard procedures. Body mass index (BMI) was calculated as weight in kilograms divided by height in meters squared (kg/m²) and categorized using standard WHO-based BMI criteria [13].

Glycemic control was assessed using the most recent HbA1c measurement available within 3 months before participant assessment. HbA1c ≥7.0% was defined as poor glycemic control, whereas HbA1c <7.0% was considered adequate glycemic control. This threshold was based on the American Diabetes Association recommendation that an HbA1c target of <7% is appropriate for most nonpregnant adults with diabetes [14].

Serum 25(OH)D measurements were retrieved from hospital laboratory records. Serum 25(OH)D was measured using an enzyme-linked immunosorbent assay (ELISA) according to the laboratory's standard operating procedure. Vitamin D deficiency was defined a priori as a serum 25(OH)D concentration <20 ng/mL, whereas concentrations ≥20 ng/mL were categorized as non-deficient [15].

The HbA1c and serum 25(OH)D results were obtained from routine clinical laboratory records and were not necessarily measured on the same date. The most recent eligible HbA1c result within the preceding 3 months and the available serum 25(OH)D result during the study period were used for analysis. Because a standardized interval between the two laboratory measurements was not specified in the clinical records, an exact measurement interval could not be established for all participants. This potential variation in measurement timing was considered when interpreting the observed association.

Study variables and operational definitions

The dependent variable was glycemic control status. HbA1c <7.0% was categorized as adequate glycemic control and HbA1c ≥7.0% as poor glycemic control. The primary independent variable was vitamin D status, categorized according to serum 25(OH)D concentration as vitamin D deficient (<20 ng/mL) or non-deficient (≥20 ng/mL) [15]. For regression analysis, vitamin D non-deficiency (≥20 ng/mL) was used as the reference category.

Other variables assessed included age, sex, marital status, education, employment status, monthly household income, BMI, duration of diabetes, family history of diabetes, smoking, physical activity, dietary adherence, medication adherence, type of antidiabetic treatment, hypertension, dyslipidemia, chronic kidney disease, frequency of blood glucose monitoring, and previous diabetes education.

Age was categorized as <50 years and ≥50 years, BMI as <30 kg/m² and ≥30 kg/m², and diabetes duration as <10 years and ≥10 years. These categories were selected for the analysis based on clinically meaningful thresholds and were applied consistently throughout the descriptive and regression analyses. Because categorization of continuous variables may result in some loss of information, the resulting estimates should be interpreted as comparisons between the specified categories rather than as evidence of biological threshold effects.

Dietary and medication adherence

Dietary adherence and medication adherence were assessed using structured self-reported items included in the study questionnaire. Participants were classified as having good or poor dietary adherence and good or poor medication adherence according to the predefined response criteria used during data collection. The same classification criteria were applied consistently to all participants, and both variables were entered into the statistical analysis as categorical variables.

Vitamin D supplementation and sunlight exposure were also assessed through the questionnaire because of their potential relevance to vitamin D status. These variables were considered potential determinants of serum 25(OH)D concentration but were not included in the final multivariable model. Season or calendar timing of vitamin D measurement was likewise not incorporated into the final regression model.

Data collection tool

A structured and standardized questionnaire was used for data collection (Appendix). The questionnaire was developed by the research team in consultation with consultant endocrinologists, diabetes educators, and senior clinical staff involved in diabetes care. Content validity was assessed by an expert committee, and the questionnaire was pilot-tested before the main study to evaluate clarity, comprehensibility, relevance, sequence, and feasibility.

The questionnaire included sections covering sociodemographic characteristics, lifestyle behaviors, physical activity, dietary adherence, clinical history, diabetes treatment, medication adherence, and diabetes self-management practices. Clinical and laboratory information, including HbA1c and serum 25(OH)D results, was verified against participants' medical records and hospital laboratory reports.

Pilot testing of the structured questionnaire

The structured questionnaire was pilot-tested before initiation of the study on 20 eligible adult patients with type 2 diabetes mellitus who were not included in the final study sample. The purpose of the pilot testing was to assess the clarity, comprehensibility, relevance, sequence, and feasibility of the questionnaire and to identify any difficulties encountered by participants during face-to-face administration.

Feedback obtained during the pilot testing indicated that the questionnaire was generally understandable and relevant to the study objectives. Based on the feedback from the 20 participants and the observations of the investigators, the questionnaire was further refined before the main data collection. In particular, the questionnaire was reorganized into clearly defined sections to improve its logical flow and ease of administration, and appropriate measurement units were added to numerical variables to improve clarity and standardization. These included units for anthropometric measurements, laboratory parameters, and other quantitative variables, where applicable. Minor wording and formatting modifications were also made where necessary.

The pilot testing did not result in any changes to the primary study variables, exposure and outcome definitions, or analytical framework. The final revised questionnaire was considered clear, comprehensive, and feasible for use in the outpatient setting. All 20 participants involved in the pilot testing were excluded from the final study analysis.

Statistical analysis

Data were entered into Microsoft Excel (Microsoft Corp., Redmond, WA) and analyzed using IBM SPSS Statistics version 25.0 (IBM Corp., Armonk, NY). Continuous variables were assessed for normality using the Shapiro-Wilk test and were summarized as mean ± standard deviation or median (interquartile range), as appropriate. Categorical variables were presented as frequencies and percentages.

Independent-samples t-tests were used to compare normally distributed continuous variables between participants with adequate and poor glycemic control, while Pearson's chi-square tests were used for categorical variables. Univariable analyses were initially performed to examine the association between participant characteristics and poor glycemic control.

Variables with p<0.20 in univariable analysis, together with clinically relevant potential confounders, were considered for inclusion in the multivariable binary logistic regression model. Adjusted odds ratios (AORs) with 95% confidence intervals (CIs) were reported. A two-sided p<0.05 was considered statistically significant.

Poor glycemic control (HbA1c ≥7.0%) was specified as the dependent variable, and vitamin D non-deficiency (25(OH)D ≥20 ng/mL) was used as the reference category for the primary exposure. The final multivariable model included vitamin D deficiency, age ≥50 years, BMI ≥30 kg/m², diabetes duration ≥10 years, hypertension, dyslipidemia, physical inactivity, poor dietary adherence, poor medication adherence, previous diabetes education, and smoking.

Vitamin D supplementation, sunlight exposure, and season or calendar timing of vitamin D measurement were not included in the final multivariable model. Accordingly, the possibility of residual confounding by these factors was considered when interpreting the adjusted association between vitamin D deficiency and poor glycemic control.

Ethical considerations

The study received ethical approval from the Institutional Review Board of Lady Reading Hospital Medical Teaching Institution (LRH MTI), Peshawar (Ref. No. 1287/LRH/MTI, dated 13/02/2024) before initiation of the study. Written informed consent was obtained from all participants before enrollment. Participation was voluntary, and participants were informed that they could withdraw from the study at any time without affecting their medical care. Participant confidentiality was maintained throughout the study. Personal identifiers were not used in the analytical dataset; unique study identification codes were assigned to participants, and access to study data was restricted to the research team.

## Results

Among the 350 participants, the largest age group was 50-59 years, comprising 116 (33.14%) participants, followed by those aged ≥60 years with 88 (25.14%). Men accounted for 183 (52.29%) participants and females for 167 (47.71%). Most participants were married (n=297; 84.86%), while 110 (31.43%) had secondary education and 176 (50.29%) were unemployed or retired. Regarding clinical characteristics, 144 (41.14%) participants had BMI ≥30 kg/m², 136 (38.86%) had diabetes duration ≥10 years, and hypertension was present in 214 (61.14%) participants (Table 1).

**Table 1:** Sociodemographic, Clinical, Treatment, and Self-Management Characteristics of Study Participants. Values are presented as frequency and percentage, n (%). Percentages are based on the total study population (N = 350).

Variable	Category	Frequency, n (%)
Age (years)	18–39	56 (16.00)
	40–49	90 (25.71)
	50–59	116 (33.14)
	≥60	88 (25.14)
Sex	Male	183 (52.29)
	Female	167 (47.71)
Marital status	Married	297 (84.86)
	Single/Widowed/Divorced	53 (15.14)
Educational level	No formal education	62 (17.71)
	Primary	72 (20.57)
	Secondary	110 (31.43)
	Higher	106 (30.29)
Employment status	Employed	174 (49.71)
	Unemployed/Retired	176 (50.29)
Monthly household income	Low	128 (36.57)
	Middle	154 (44.00)
	High	68 (19.43)
BMI (kg/m²)	<30	206 (58.86)
	≥30	144 (41.14)
Duration of diabetes	<10 years	214 (61.14)
	≥10 years	136 (38.86)
Hypertension	Yes	214 (61.14)
	No	136 (38.86)
Dyslipidemia	Yes	168 (48.00)
	No	182 (52.00)
Family history of diabetes	Yes	198 (56.57)
	No	152 (43.43)
Diabetes-related complications	Yes	162 (46.29)
	No	188 (53.71)
Neuropathy	Yes	92 (26.29)
	No	258 (73.71)
Smoking	Yes	71 (20.29)
	No	279 (79.71)
Type of antidiabetic therapy	Oral drugs only	210 (60.00)
	Insulin only	42 (12.00)
	Oral drugs + insulin	98 (28.00)
Medication adherence	Poor	122 (34.86)
	Good	228 (65.14)
Dietary adherence	Poor	180 (51.43)
	Good	170 (48.57)
Physical activity	Physically inactive	194 (55.43)
	Physically active	156 (44.57)
Previous diabetes education	No	149 (42.57)
	Yes	201 (57.43)
Blood glucose monitoring	Daily	86 (24.57)
	Weekly	101 (28.86)
	Monthly	92 (26.29)
	Rarely/Never	71 (20.29)

Vitamin D deficiency was identified in 218 (62.29%) participants, whereas 132 (37.71%) were non-deficient. Overall, poor glycemic control (HbA1c ≥7.0%) was observed in 226 (64.57%) participants, while 124 (35.43%) had adequate glycemic control (Table 2).

**Table 2:** Vitamin D Status and Glycemic Control Among Study Participants (N = 350)

Variable	Category	Frequency (n)	Percentage (%)
Vitamin D status	Deficient (<20 ng/mL)	218	62.29
	Non-deficient (≥20 ng/mL)	132	37.71
Glycemic control	Poor (HbA1c ≥7.0%)	226	64.57
	Adequate (HbA1c <7.0%)	124	35.43

Participants with poor glycemic control had significantly lower mean serum 25(OH)D levels than those with adequate control (16.72 ± 6.43 vs. 23.84 ± 7.16 ng/mL; t = 9.47, p <0.001). Vitamin D deficiency was also more frequent among participants with poor glycemic control, occurring in 164 (72.57%) compared with 54 (43.55%) participants with adequate control (χ² = 29.48, p <0.001). Poor-control participants also had higher mean HbA1c, BMI, diabetes duration, and age (Table 3). 

**Table 3:** Serum 25(OH)D and Glycemic Parameters According to Glycemic Control Status

Variable	Adequate Glycemic Control (n=124)	Poor Glycemic Control (n=226)	Test value	p-value
Serum 25(OH)D (ng/mL), mean ± SD	23.84 ± 7.16	16.72 ± 6.43	*t* = 9.47	<0.001
Vitamin D deficiency, n (%)	54 (43.55)	164 (72.57)	χ² = 29.48	<0.001
Vitamin D non-deficiency, n (%)	70 (56.45)	62 (27.43)		
HbA1c (%), mean ± SD	6.39 ± 0.41	8.94 ± 1.48	*t* = −18.88	<0.001
BMI (kg/m²), mean ± SD	27.08 ± 3.41	30.41 ± 4.63	*t* = −7.12	<0.001
Diabetes duration (years), mean ± SD	6.42 ± 3.58	10.21 ± 5.07	*t* = −7.98	<0.001
Age (years), mean ± SD	51.24 ± 10.31	55.87 ± 9.92	*t* = −4.21	<0.001

Several participant characteristics were significantly associated with poor glycemic control on unadjusted analysis. A total of 158 (69.91%) participants aged ≥50 years demonstrated poor control compared with 46 (37.10%) with adequate control (χ^2^ = 36.42, p <0.001). Similarly, BMI ≥30 kg/m², diabetes duration ≥10 years, hypertension, dyslipidemia, smoking, physical inactivity, poor dietary adherence, poor medication adherence, and no previous diabetes education were significantly associated with poor glycemic control. Insulin therapy was not significantly associated with poor control (p = 0.081), while sex also showed no significant association (p = 0.412) (Table 4).

**Table 4:** Unadjusted Associations Between Participant Characteristics and Poor Glycemic Control Values are presented as frequency and percentage, n (%), within each glycemic-control group. Pearson’s chi-square test was used for categorical variables.

Variable	Poor Control n (%)	Adequate Control n (%)	Test value	p-value
Age ≥50 years	158 (69.91)	46 (37.10)	χ² = 36.42	<0.001
Female sex	104 (46.02)	63 (50.81)	χ² = 0.67	0.412
BMI ≥30 kg/m²	118 (52.21)	26 (20.97)	χ² = 34.87	<0.001
Diabetes duration ≥10 years	112 (49.56)	24 (19.35)	χ² = 32.41	<0.001
Hypertension	152 (67.26)	62 (50.00)	χ² = 9.54	0.002
Dyslipidemia	125 (55.31)	43 (34.68)	χ² = 14.62	<0.001
Smoking	58 (25.66)	13 (10.48)	χ² = 10.88	0.001
Physical inactivity	150 (66.37)	44 (35.48)	χ² = 32.61	<0.001
Poor dietary adherence	144 (63.72)	36 (29.03)	χ² = 42.76	<0.001
Poor medication adherence	103 (45.58)	19 (15.32)	χ² = 32.74	<0.001
No diabetes education	116 (51.33)	33 (26.61)	χ² = 21.87	<0.001
Insulin therapy	98 (43.36)	42 (33.87)	χ² = 3.05	0.081
Vitamin D deficiency	164 (72.57)	54 (43.55)	χ² = 29.48	<0.001

In unadjusted logistic regression, vitamin D deficiency was significantly associated with poor glycemic control. Participants with vitamin D deficiency had 3.44 times higher odds of poor glycemic control than vitamin D non-deficient participants (OR = 3.44, 95% CI: 2.18-5.43, p <0.001) (Table 5).

**Table 5:** Unadjusted Association Between Vitamin D Deficiency and Poor Glycemic Control OR = odds ratio; CI = confidence interval. The unadjusted association was assessed using binary logistic regression

Vitamin D Status	Poor Glycemic Control n (%)	Adequate Glycemic Control n (%)	OR	95% CI	p-value
Deficient (<20 ng/mL)	164 (72.57)	54 (43.55)	3.44	2.18-5.43	<0.001
Non-deficient (≥20 ng/mL)	62 (27.43)	70 (56.45)	1.00	Reference	

After adjustment for potential demographic, clinical, lifestyle, and treatment-related confounders, vitamin D deficiency remained independently associated with poor glycemic control (AOR = 2.87, 95% CI: 1.76-4.68, p <0.001). Other independent factors included age ≥50 years (AOR = 1.78, p = 0.019), BMI ≥30 kg/m² (AOR = 2.41, p <0.001), diabetes duration ≥10 years (AOR = 2.12, p = 0.002), dyslipidemia (AOR = 1.66, p = 0.031), physical inactivity (AOR = 2.18, p = 0.002), poor dietary adherence (AOR = 2.63, p <0.001), poor medication adherence (AOR = 3.21, p <0.001), and lack of previous diabetes education (AOR = 1.69, p = 0.031). Hypertension and smoking were not independently significant (Table 6).

**Table 6:** Multivariable Logistic Regression Analysis of Factors Associated With Poor Glycemic Control AOR = adjusted odds ratio; CI = confidence interval. Poor glycemic control (HbA1c ≥7.0%) was the dependent variable. Variables with p <0.20 in univariable analysis, together with clinically relevant potential confounders, were considered for inclusion in the multivariable logistic regression model.

Variable	Adjusted Odds Ratio (AOR)	95% CI	p-value
Vitamin D deficiency (<20 ng/mL)	2.87	1.76-4.68	<0.001
Age ≥50 years	1.78	1.10-2.89	0.019
BMI ≥30 kg/m²	2.41	1.46-3.96	<0.001
Diabetes duration ≥10 years	2.12	1.31-3.43	0.002
Hypertension	1.48	0.94-2.32	0.091
Dyslipidemia	1.66	1.05-2.63	0.031
Physical inactivity	2.18	1.34-3.55	0.002
Poor dietary adherence	2.63	1.61-4.29	<0.001
Poor medication adherence	3.21	1.89-5.45	<0.001
No previous diabetes education	1.69	1.05-2.71	0.031
Smoking	1.54	0.92-2.59	0.101

## Discussion

The present study revealed a high prevalence of vitamin D deficiency (62.29%) and poor glycemic control (64.57%) among adults with T2DM. The prevalence of vitamin D deficiency observed in our study was comparable to that reported in other populations with T2DM. One study in Saudi Arabia found that 60.8% of patients with T2DM were vitamin D deficient and reported a statistically significant correlation between vitamin D level and glycemic control [16]. In a large cross-sectional study of 1,074 patients with T2DM, Wang et al. (2023) observed that 65.0% had inadequate vitamin D levels, with a mean 25(OH)D concentration of 17.05 ng/mL [17]. The comparable prevalence observed in our Pakistani population suggests that vitamin D deficiency is also common among adults with T2DM in this setting, although differences in geographical location, population characteristics, sunlight exposure, dietary patterns, and clinical characteristics should be considered when comparing prevalence estimates across studies.

One of the most important observed differences in the present study was the lower serum 25(OH)D level among individuals with poor glycemic control (16.72 ± 6.43 ng/mL) compared with those with adequate control (23.84 ± 7.16 ng/mL; p <0.001). Vitamin D deficiency was present in 72.57% (n=164) of participants with poor glycemic control, compared with 43.55% (54) of those with adequate control (χ² = 29.48, p <0.001). These findings are consistent with Iqbal et al. (2016), who observed that 58.7% of Pakistani patients with poor glycemic control and 30.6% of those with good control were vitamin D deficient (p = 0.006) [12]. Likewise, Wang et al. (2023) reported higher HbA1c levels among patients with vitamin D deficiency and an inverse correlation between 25(OH)D and HbA1c (r = -0.119, p <0.001) [17]. Although these findings demonstrate a consistent observational association, they should not be interpreted as evidence that vitamin D deficiency causes poor glycemic control.

Logistic regression was used to further quantify the association between vitamin D deficiency and poor glycemic control. In the unadjusted analysis, participants with vitamin D deficiency had higher odds of poor glycemic control than those without vitamin D deficiency (OR = 3.44, 95% CI: 2.18-5.43; p <0.001). This association remained statistically significant after adjustment for demographic, clinical, lifestyle, and treatment-related factors (AOR = 2.87, 95% CI: 1.76-4.68; p <0.001), indicating higher adjusted odds of poor glycemic control among vitamin D-deficient participants. This finding is consistent with the study by Iqbal et al. (2016) in Pakistan, which reported an adjusted OR of 4.86 (95% CI: 1.9-11.9; p <0.001) for the association between vitamin D deficiency and poor glycemic control [12]. Similarly, a Saudi study reported that vitamin D deficiency remained independently associated with poor glycemic control after adjustment for clinical and treatment-related factors [16]. The consistency of the direction of association across these observational studies supports further investigation, while differences in effect estimates may reflect variation in study populations, definitions, clinical characteristics, and potential residual confounding.

Other clinical and metabolic characteristics in our study also demonstrated clinically relevant associations. Participants with poor glycemic control had higher BMI (30.41 ± 4.63 vs. 27.08 ± 3.41 kg/m²), longer diabetes duration (10.21 ± 5.07 vs. 6.42 ± 3.58 years), and older age (55.87 ± 9.92 vs. 51.24 ± 10.31 years), all with p <0.001. Obesity (AOR = 2.41, p <0.001) and diabetes duration ≥10 years (AOR = 2.12, p = 0.002) were independently associated with poor glycemic control in the multivariable analysis. These findings are consistent with evidence indicating that obesity and longer diabetes duration may be associated with poorer metabolic control [7,18].

Importantly, the multivariable analysis also demonstrated associations between poor glycemic control and several modifiable behavioural and treatment-related factors. Poor medication adherence (AOR = 3.21, p <0.001), poor dietary adherence (AOR = 2.63, p <0.001), physical inactivity (AOR = 2.18, p = 0.002), and lack of previous diabetes education (AOR = 1.69, p = 0.031) were independently associated with higher odds of poor glycemic control. These findings emphasize that glycemic control in T2DM is multifactorial and may reflect a combination of treatment adherence, dietary behaviour, physical activity, diabetes education, disease duration, adiposity, and other clinical characteristics. Dyslipidemia was also independently associated with poor glycemic control (AOR = 1.66, p = 0.031). The persistence of the association between vitamin D deficiency and poor glycemic control after adjustment for these factors suggests that the observed relationship was not fully explained by the measured behavioural and clinical characteristics; however, residual confounding cannot be excluded.

The current results suggest a possible biological association between vitamin D status and glucose regulation but do not imply causality because of the cross-sectional study design. The observed association is biologically plausible because vitamin D has been proposed to influence pancreatic β-cell function, insulin secretion, and insulin sensitivity. Importantly, intervention evidence is also suggestive: in a meta-analysis of 39 randomized controlled trials involving 2,982 participants, Chen et al. (2024) reported a significant reduction in HbA1c following vitamin D supplementation (WMD = −0.30%, 95% CI: −0.43 to −0.18), together with an improvement in HOMA-IR [19]. However, previous studies have reported conflicting findings, and a large U.S. study found no adjusted association between vitamin D deficiency and poor glycemic control [20]. These differences indicate that the relationship may vary according to population characteristics, baseline vitamin D status, diabetes-related factors, supplementation, lifestyle factors, and other sources of confounding. Therefore, the present findings support an association between vitamin D deficiency and poor glycemic control rather than establishing a therapeutic effect of vitamin D supplementation.

The findings indicate that vitamin D deficiency and poor glycemic control are both common among the studied adults with T2DM and that vitamin D deficiency was associated with higher odds of poor glycemic control after adjustment for several measured covariates. At the same time, the associations observed for physical inactivity, dietary and medication adherence, diabetes education, obesity, and diabetes duration demonstrate the multifactorial nature of glycemic control. Prospective cohort studies and appropriately designed randomized trials in Pakistani populations are warranted to clarify temporal relationships and determine whether correction of vitamin D deficiency has a clinically meaningful effect on glycemic control.

Study strengths and limitations

The strengths of this study include the use of objective laboratory measurements of serum 25(OH)D and HbA1c, clearly defined operational criteria for vitamin D deficiency and poor glycemic control, consecutive recruitment of eligible participants, standardized data collection procedures, and pilot testing of the study questionnaire. The study also evaluated a broad range of demographic, clinical, lifestyle, and treatment-related factors and used multivariable logistic regression to account for several measured potential confounders.

However, several limitations should be considered. The cross-sectional design does not allow temporal or causal relationships to be established between vitamin D deficiency and poor glycemic control. The study was conducted at a single center using non-probability sampling; therefore, the findings may not be generalizable to the broader Pakistani population with T2DM. Several behavioural variables, including dietary adherence, medication adherence, and physical activity, were self-reported and may be subject to recall or reporting bias. Although sunlight exposure and vitamin D supplementation were collected, they were not included in the final multivariable model, and seasonal or calendar timing of vitamin D measurement was not adjusted for. Dietary vitamin D intake was also not fully evaluated. These factors may have contributed to residual confounding. In addition, HbA1c and serum 25(OH)D measurements were not necessarily obtained on the same date, and a standardized interval between the two measurements could not be established for all participants. Another limitation is the requirement for both a recent HbA1c result and an available serum 25(OH)D measurement for inclusion. Patients who underwent vitamin D testing may differ systematically from the broader outpatient T2DM population in terms of clinical characteristics, healthcare utilization, supplementation, or other factors. Therefore, selection bias cannot be excluded, and the findings should primarily be generalized to adults with T2DM with characteristics and healthcare access comparable to the study population. Finally, categorization of variables such as age, BMI, and diabetes duration may result in some loss of information compared with analysis of continuous measurements.

## Conclusions

A majority of the adults with T2DM were also vitamin D deficient, and there was a significant association between vitamin D deficiency and poor glycemic control. Patients with poor glycemic control had reduced vitamin D levels and an increased risk of vitamin D deficiency compared with those with adequate glycemic control. This association persisted after adjusting for relevant demographics, clinical, lifestyle, and treatment-related factors, indicating that vitamin D status could be an important factor associated with glycemic management for patients with T2DM. The cross-sectional design of the study, however, does not allow for causal statements. Further multicenter prospective and interventional studies are recommended to clarify the temporal relationship and determine whether correction of vitamin D deficiency can contribute to improved glycemic control.

## Appendices

A structured questionnaire to investigate the association between vitamin D deficiency and poor glycemic control in patients with type 2 diabetes mellitus was distributed to the patient population (Table 7).

**Table 7:** Structured Questionnaire for Association Between Vitamin D Deficiency and Poor Glycemic Control in Patients With Type 2 Diabetes Mellitus

Section / Item	Question / Variable	Response / Recording Field
Participant Identification	Participant ID	______________________________
	Date of Interview	____ / ____ / ______
	Interviewer ID	______________________________
Instructions for Interviewer	Administration	Complete the questionnaire through a face-to-face interview. Clinical and laboratory information should be verified from the patient's medical record and hospital laboratory reports wherever applicable. Do not record the participant's name.
A. Eligibility Screening	A1. Age	______ years
	A2. Age ≥18 years?	☐ Yes ☐ No
	A3. Confirmed type 2 diabetes mellitus?	☐ Yes ☐ No
	A4. Duration of T2DM	______ years
	A5. T2DM duration ≥6 months?	☐ Yes ☐ No
	A6. HbA1c measured within the preceding 3 months?	☐ Yes ☐ No
	A7. Serum 25(OH)D measurement available?	☐ Yes ☐ No
	A8. Pregnant?	☐ Yes ☐ No
	A9. Type 1 diabetes mellitus?	☐ Yes ☐ No
	A10. Gestational diabetes or other type of diabetes?	☐ Yes ☐ No
	A11. Acute diabetic emergency or severe illness requiring hospitalization?	☐ Yes ☐ No
	A12. Cognitive impairment or severe psychiatric disorder preventing a reliable interview?	☐ Yes ☐ No
	A13. Incomplete medical record?	☐ Yes ☐ No
	Eligibility status	☐ Eligible ☐ Not eligible
B. Sociodemographic Characteristics	B1. Age	______ years
	Age category for analysis	☐ <50 years ☐ ≥50 years
	B2. Sex	☐ Male ☐ Female
	B3. Marital status	☐ Married ☐ Single ☐ Widowed ☐ Divorced
	B4. Educational level	☐ No formal education ☐ Primary ☐ Secondary ☐ Higher
	B5. Employment status	☐ Employed ☐ Unemployed ☐ Retired ☐ Other: __________
	Employment category for analysis	☐ Employed ☐ Unemployed/Retired
	B6. Monthly household income	☐ Low ☐ Middle ☐ High
C. Anthropometric Measurements	C1. Weight	______ kg
	C2. Height	______ m
	C3. BMI	______ kg/m²
	C4. BMI category for analysis	☐ <30 kg/m² ☐ ≥30 kg/m²
D. Diabetes History	D1. Duration of T2DM	______ years
	D2. Diabetes duration category	☐ <10 years ☐ ≥10 years
	D3. Family history of diabetes	☐ Yes ☐ No ☐ Don't know
	D4. Diabetes-related complications diagnosed by a doctor	☐ Yes ☐ No ☐ Don't know
	D5. Diabetic neuropathy diagnosed by a doctor	☐ Yes ☐ No ☐ Don't know
E. Comorbidities	E1. Hypertension diagnosed by a healthcare professional	☐ Yes ☐ No
	E2. Dyslipidemia/high cholesterol or abnormal blood lipids	☐ Yes ☐ No
	E3. Chronic kidney disease	☐ Yes ☐ No ☐ Don't know
F. Smoking Status	F1. Current tobacco smoking	☐ Yes ☐ No
	F2. Type of tobacco product, if applicable	☐ Cigarettes ☐ Cigars ☐ Naswar ☐ Other: __________
	F3. Duration of smoking	______ years
	F4. Approximate amount consumed	______ cigarettes/units per day
	Primary analysis variable	☐ Smoker ☐ Non-smoker
G. Physical Activity	G1. Days per week with ≥30 minutes of physical activity/exercise during the past 4 weeks	☐ 0 days ☐ 1–2 days ☐ 3–4 days ☐ ≥5 days
	G2. Usual type of physical activity	☐ Walking ☐ Running/jogging ☐ Cycling ☐ Household/farm work ☐ Gym/exercise ☐ Other: ______ ☐ None
	G3. Average duration on an active day	☐ <30 min ☐ 30–59 min ☐ ≥60 min
	Physical activity classification	☐ Physically active ☐ Physically inactive
	Classification criterion	Physically active = regular activity ≥30 minutes on ≥5 days/week
H. Dietary Adherence	H1. Frequency of following the diet recommended by doctor/dietitian	☐ Always ☐ Usually ☐ Sometimes ☐ Rarely ☐ Never
	H2. Frequency of limiting sugary foods/drinks as advised	☐ Always ☐ Usually ☐ Sometimes ☐ Rarely ☐ Never
	H3. Frequency of following recommended portion sizes	☐ Always ☐ Usually ☐ Sometimes ☐ Rarely ☐ Never
	H4. Frequency of following the recommended meal plan	☐ Always ☐ Usually ☐ Sometimes ☐ Rarely ☐ Never
	Dietary adherence classification	☐ Good ☐ Poor
	Scoring used during data collection	Always = 4; Usually = 3; Sometimes = 2; Rarely = 1; Never = 0
I. Medication Adherence	I1. In the past 7 days, how often did you miss your diabetes medication?	☐ Never ☐ Once ☐ 2–3 times ☐ 4–5 times ☐ >5 times
	I2. Do you sometimes forget to take your diabetes medication?	☐ Never ☐ Sometimes ☐ Often ☐ Always
	I3. Do you ever stop taking medication when you feel your blood glucose is controlled?	☐ No ☐ Yes
	I4. Have you ever reduced/stopped medication without consulting your doctor?	☐ No ☐ Yes
	I5. General medication adherence	☐ Good ☐ Poor
	Final medication adherence classification	☐ Good ☐ Poor
J. Diabetes Treatment	J1. Current antidiabetic treatment	☐ Oral antidiabetic drugs only ☐ Insulin only ☐ Oral antidiabetic drugs + insulin
	J2. Oral antidiabetic medication(s), if applicable	______________________________
	J3. Insulin, if applicable	______________________________
	J4. Duration of current treatment	______ years/months
K. Diabetes Education	K1. Previous formal diabetes education from a healthcare professional	☐ Yes ☐ No
	K2. Topics discussed, if applicable	☐ Blood glucose monitoring ☐ Medication use ☐ Diet/nutrition ☐ Physical activity ☐ Hypoglycemia recognition ☐ Foot care ☐ Diabetes complications ☐ Other: ______
	K3. Time since last diabetes education	☐ Within last 6 months ☐ 6–12 months ago ☐ >1 year ago ☐ Don't remember
	Primary analysis variable	☐ Previous diabetes education: Yes ☐ Previous diabetes education: No
L. Blood Glucose Monitoring	L1. Frequency of home blood glucose monitoring	☐ Daily ☐ Weekly ☐ Monthly ☐ Rarely/Never
	L2. Usual monitoring method	☐ Glucometer ☐ Continuous glucose monitoring system ☐ Laboratory testing only ☐ Other: ______
M. Vitamin D-Related Information	M1. Previous diagnosis of vitamin D deficiency	☐ Yes ☐ No ☐ Don't know
	M2. Currently taking vitamin D supplements	☐ Yes ☐ No
	M3. Frequency of supplementation, if applicable	☐ Daily ☐ Weekly ☐ Monthly ☐ Irregularly
	M4. Duration of vitamin D supplementation	______ months
	M5. Vitamin D treatment during the preceding 3 months	☐ Yes ☐ No ☐ Don't know
N. Sunlight Exposure	N1. Typical daily outdoor exposure during daylight	☐ <15 min ☐ 15–30 min ☐ 31–60 min ☐ >60 min
	N2. Days per week spent outdoors during daylight	☐ 0–1 days ☐ 2–3 days ☐ 4–5 days ☐ 6–7 days
	N3. Arms/legs usually exposed during outdoor exposure	☐ Yes ☐ No ☐ Sometimes
O. Laboratory and Clinical Record Form	Serum 25(OH)D	______ ng/mL
	Vitamin D status	☐ Deficient <20 ng/mL ☐ Non-deficient ≥20 ng/mL
	HbA1c	______ %
	Glycemic control	☐ Poor ≥7.0% ☐ Adequate <7.0%
	Fasting blood glucose, if available	______ mg/dL
	Serum creatinine, if available	______ mg/dL
	eGFR, if available	______ mL/min/1.73m²
	Total cholesterol, if available	______ mg/dL
	LDL-C, if available	______ mg/dL
	HDL-C, if available	______ mg/dL
	Triglycerides, if available	______ mg/dL
	25(OH)D measurement date	____ / ____ / ______
	HbA1c measurement date	____ / ____ / ______
P. Final Outcome Classification	Primary exposure: Vitamin D status	☐ Vitamin D deficient: 25(OH)D <20 ng/mL ☐ Vitamin D non-deficient: 25(OH)D ≥20 ng/mL
	Primary outcome: Glycemic control	☐ Poor glycemic control: HbA1c ≥7.0% ☐ Adequate glycemic control: HbA1c <7.0%
Final Verification	Medical record/laboratory information verified	☐ Yes ☐ No
	Questionnaire completed	☐ Yes ☐ No
	Investigator initials	__________________
